# Supplementary material for: Footprints of antigen processing boost MHC class II natural ligand predictions
Source: Genome Med. 2018 Nov 16;10:84. doi: 10.1186/s13073-018-0594-6 (PMC6240193; doi:10.1186/s13073-018-0594-6)
Supplement: Supplementary file 1 — Figure S1. Binding preferences learnt by each individual neural network. Figure S2. Pearson correlation coefficient (PCC) heatmaps comparing core motifs learnt by the single EL and BA models. Table S1. Standard deviations for Figure S2. Figure S3. Binding preferences learned by the combined NNAlign models. Figure S4. Processing signals located at N and C terminal regions. Figure S5. Heatmap representation of the processing signal’s Position-Specific Scoring Matrix (PSSM). Figure S6. Correlation between processing signals found in all six data sets. Figure S7. Processing signal learned by the NNAlign model trained on DR15 Pm data set. (PDF 3717 kb) [file 13073_2018_594_MOESM1_ESM.pdf]

**A**

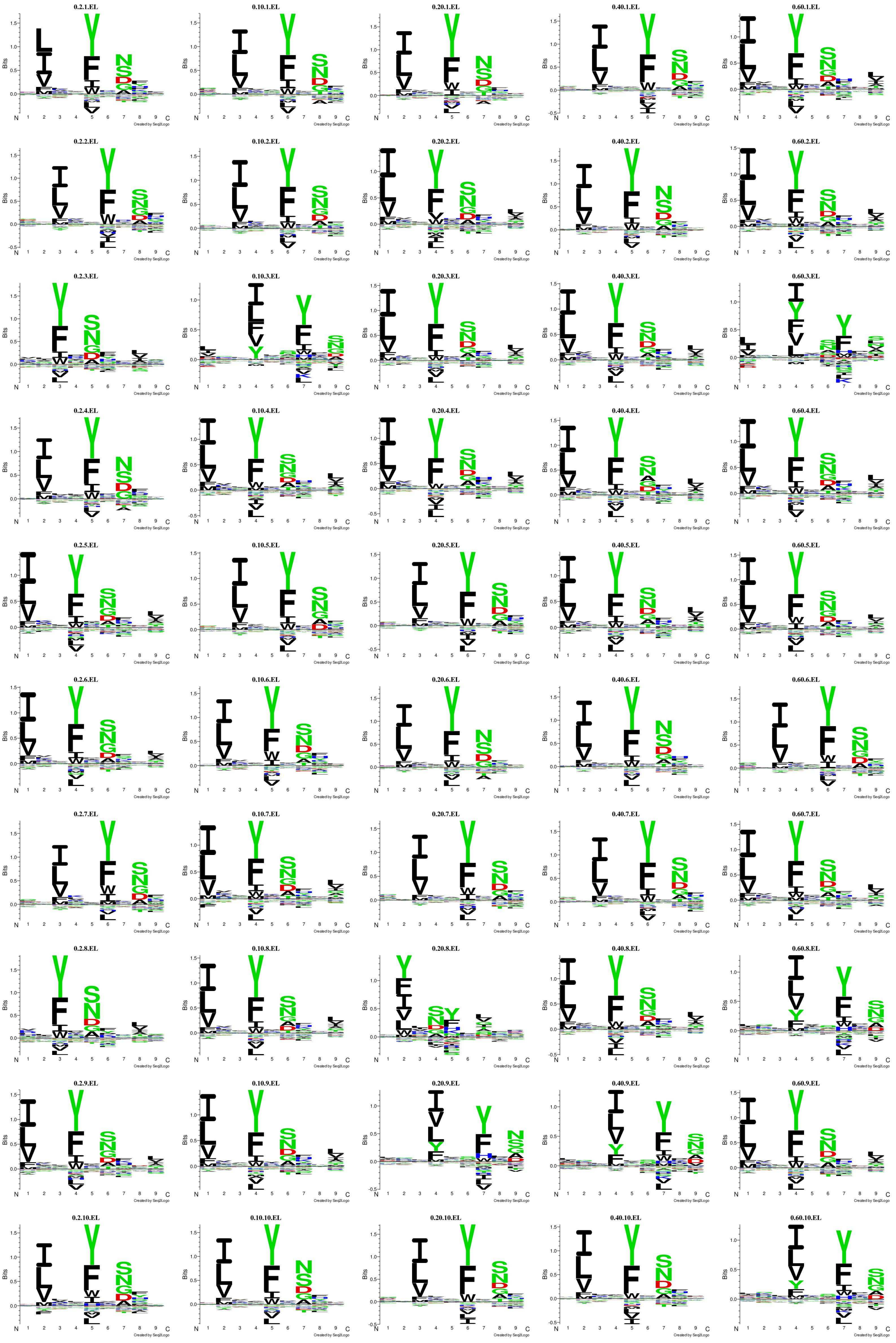
0.2.1.EL
0.10.1.EL
0.20.1.EL
0.40.1.EL
0.60.1.EL
0.2.2.EL
0.10.2.EL
0.20.2.EL
0.40.2.EL
0.60.2.EL
0.2.3.EL
0.10.3.EL
0.20.3.EL
0.40.3.EL
0.60.3.EL
0.2.4.EL
0.10.4.EL
0.20.4.EL
0.40.4.EL
0.60.4.EL
0.2.5.EL
0.10.5.EL
0.20.5.EL
0.40.5.EL
0.60.5.EL
0.2.6.EL
0.10.6.EL
0.20.6.EL
0.40.6.EL
0.60.6.EL
0.2.7.EL
0.10.7.EL
0.20.7.EL
0.40.7.EL
0.60.7.EL
0.2.8.EL
0.10.8.EL
0.20.8.EL
0.40.8.EL
0.60.8.EL
0.2.9.EL
0.10.9.EL
0.20.9.EL
0.40.9.EL
0.60.9.EL
0.2.10.EL
0.10.10.EL
0.20.10.EL
0.40.10.EL
0.60.10.EL

**B**

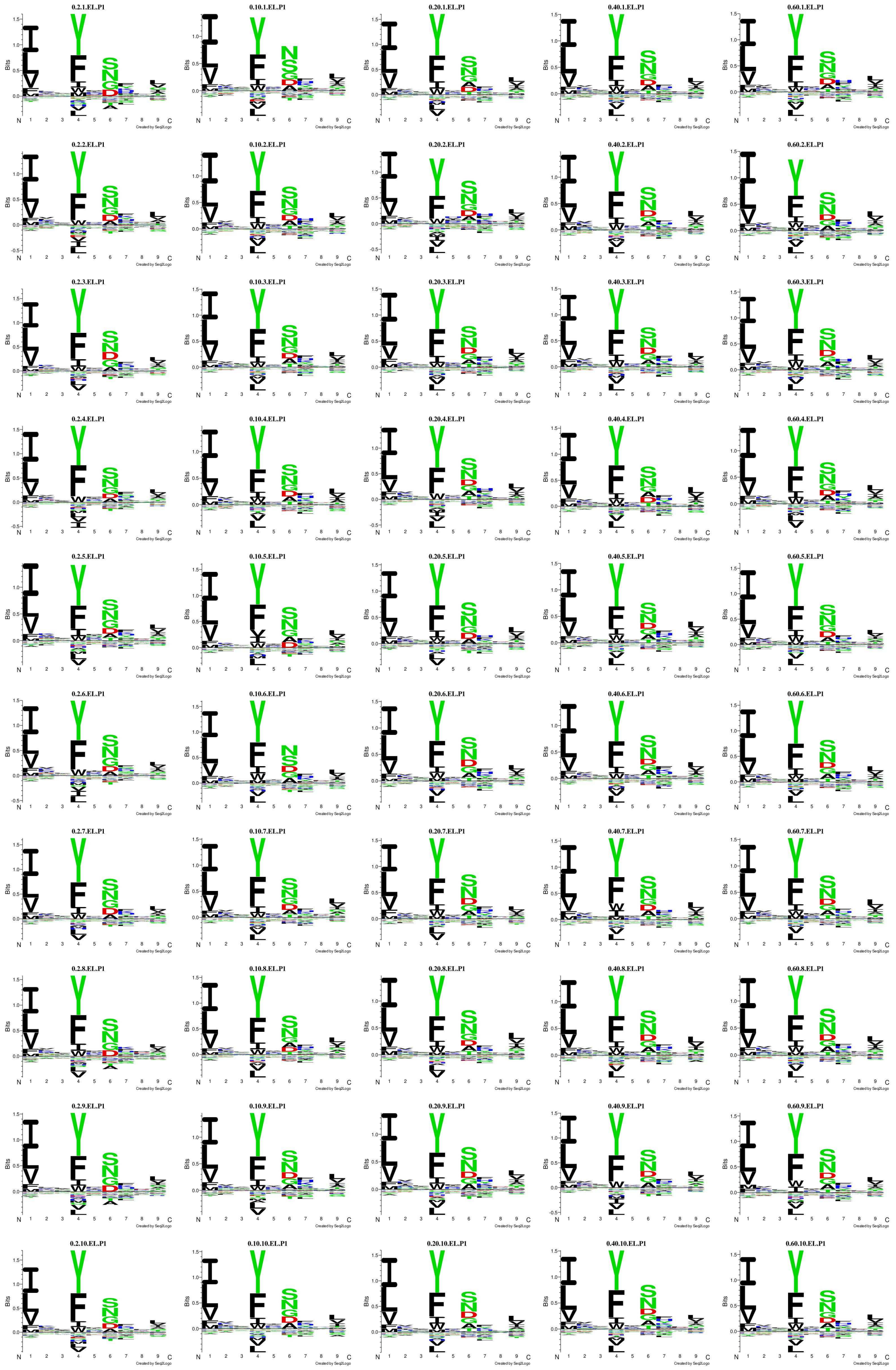


**Figure S1.** Binding preferences learned by each network in the NNAlign model trained on DR15 Ph, with and without P1 burn-in implementation (panels “B” and “A”, respectively). Each logo represents a binding core learned by the network architecture out of a total of fifty (corresponding to ten seeds, and five different hidden neuron values). Logo plots were constructed from the predicted cores in the top 1% scoring predictions of 1 million random natural peptides.

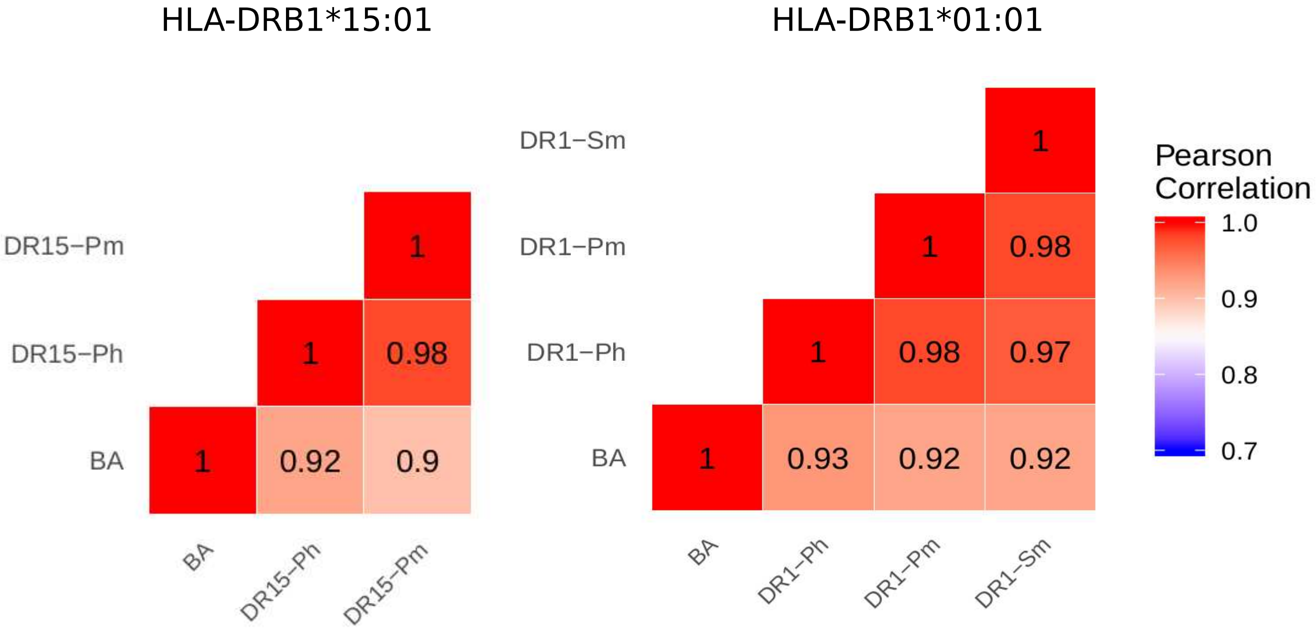


**Figure S2.** Pearson correlation coefficient (PCC) heatmaps between single EL and BA dataset core motifs. To calculate the correlation between sequence motifs, we extracted the amino acid frequencies from the motifs displayed in figure 2, and next performed a bootstrapped correlation analysis comparing the amino acid frequency values at the four anchor positions (P1, P4, P6 and P9) of the binding core between all pairs of motifs. Heatmaps report the bootstrapped PCC values; standard deviations are reported in Table S1.

| Dataset_1 | Dataset_2 | Average | STD_DEV |
|---|---|---|---|
| DR1-Ph | DR1-Pm | 0.981 | 0.009 |
| DR1-Ph | DR1-Sm | 0.968 | 0.015 |
| DR1-Pm | DR1-Sm | 0.982 | 0.009 |
| DR1-Ph | BA | 0.929 | 0.028 |
| DR1-Pm | BA | 0.923 | 0.026 |
| DR1-Sm | BA | 0.921 | 0.028 |
| DR15-Ph | DR15-Pm | 0.985 | 0.007 |
| DR15-Ph | BA | 0.926 | 0.026 |
| DR15-Pm | BA | 0.905 | 0.029 |

**Table S1.** Average Pearson correlation coefficient and standard deviations for Figure S2.

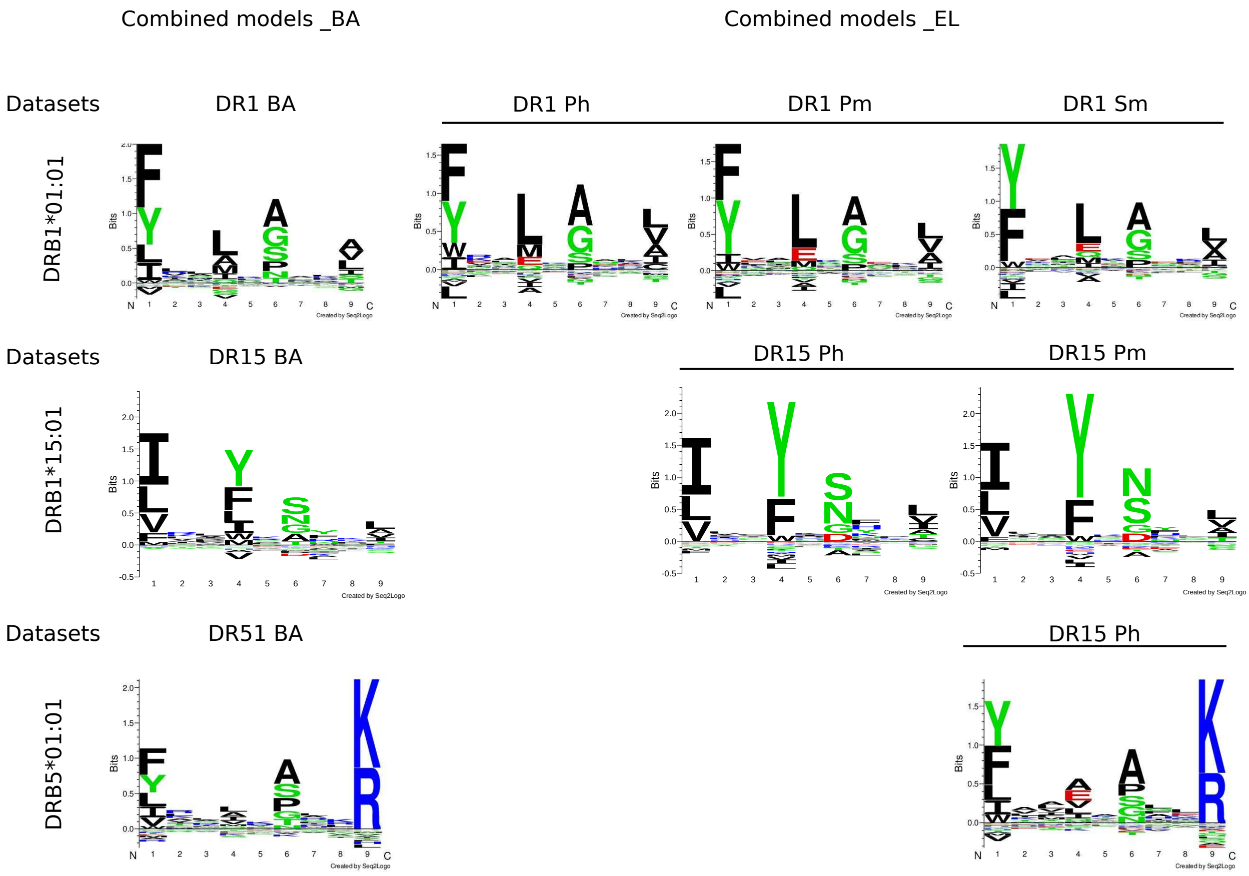


**Figure S3.** Binding preferences learned by the combined NNAlign models, trained on both Binding Affinity (BA) and Eluted Ligands (EL) data. In the top row, motifs for the DRB1*01:01 allele are shown, with overlined logoplots (right) corresponding to EL output neuron of the combined model, and the non-overlined logo (left) corresponding to the BA output neuron. Similarly, binding motifs for DRB1*15:01 are displayed in the bottom row, with overlined logos (right) also indicating the EL output neuron preference, and the non-overlined logoplot (left) indicating the BA output neuron predilection. Logos were constructed from the predicted binding cores in the top 1% scoring predictions of 900.000 random natural peptides.

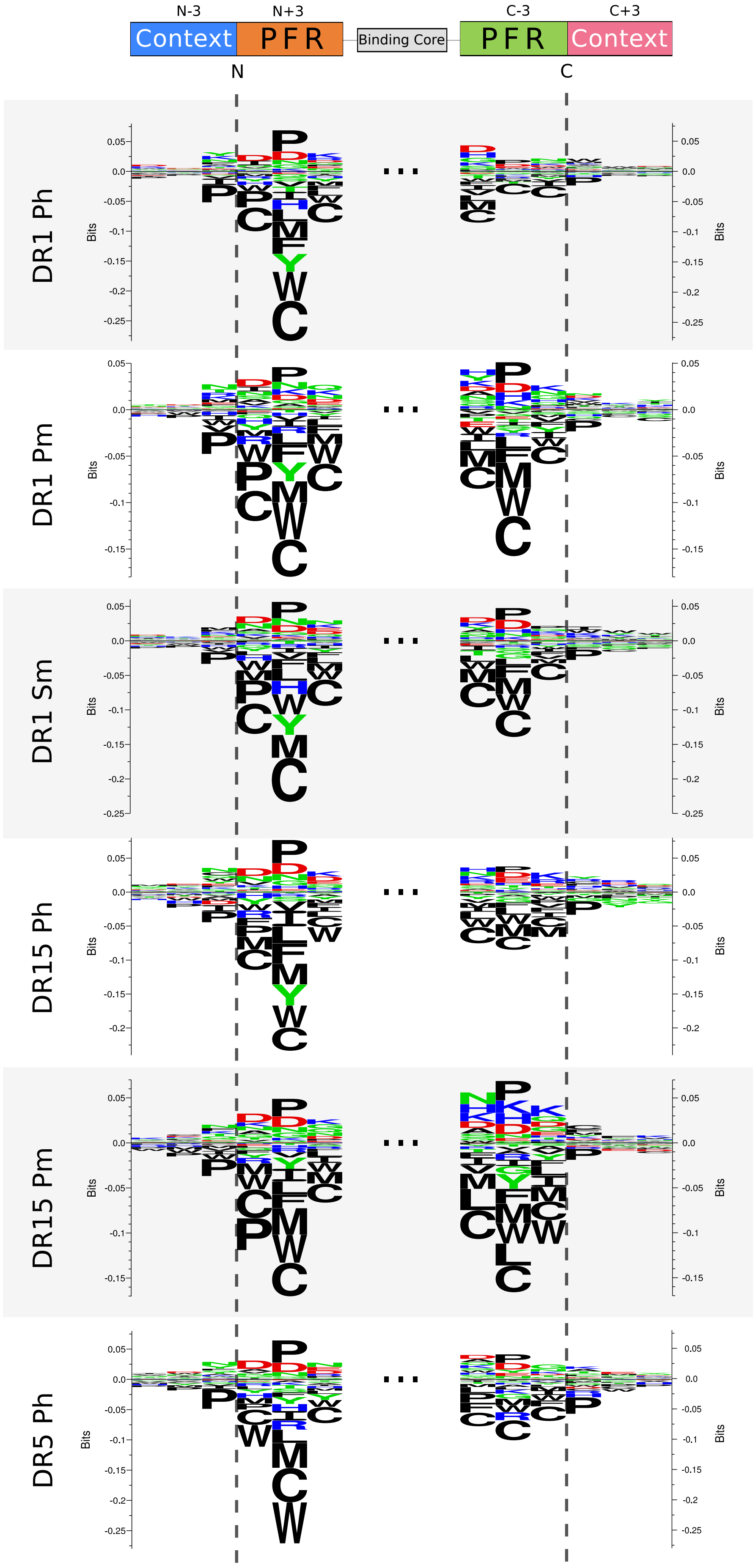


**Figure S4.** Processing signals located at N and C terminal regions for all six datasets employed in this work. For each terminus region in every dataset, we first filtered out all ligands with PFR length lower than 3. Then, we constructed the logos by selecting the closest three PFR and context residues neighboring the N and C terminus. For additional details on this processing signal construction, please refer to Figure 5.

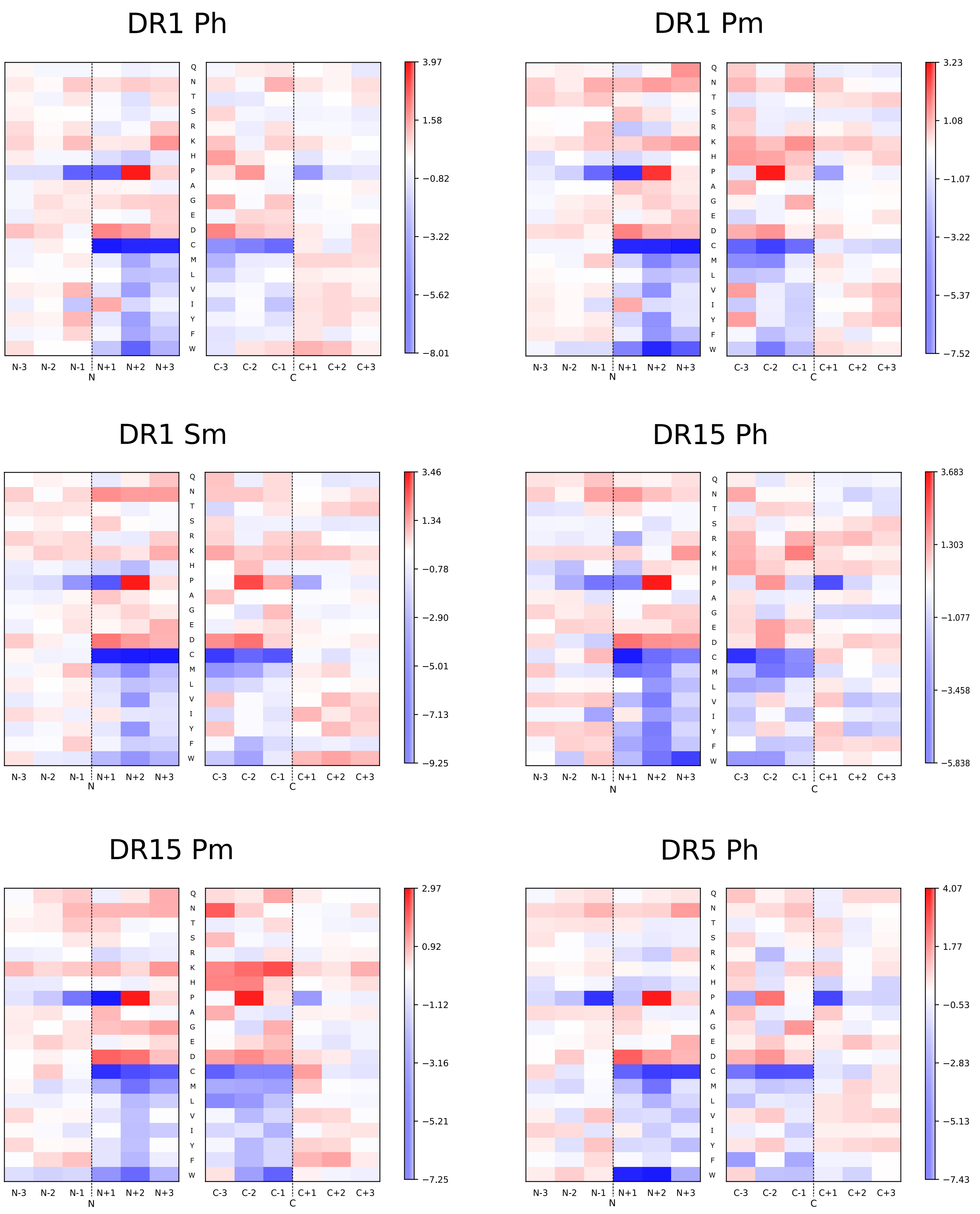


**Figure S5.** Heatmap representation of the processing signal's Position-Specific Scoring Matrix (PSSM), for all six datasets employed in this work. For each dataset, the closest three PFR and Context residues neighboring the N and C terminus are shown. Color code correspond to the information content present in each position of the PSSM, for upstream and downstream. Colorbar values indicate the power of two to which information is increased or decreased (i.e. a value of -3 indicates a 23 fold position-specific information depletion, meaning eight times more probability to find such particular depletion than the natural background frequency). For more information on how this processing signal is constructed, refer to Figure 5.

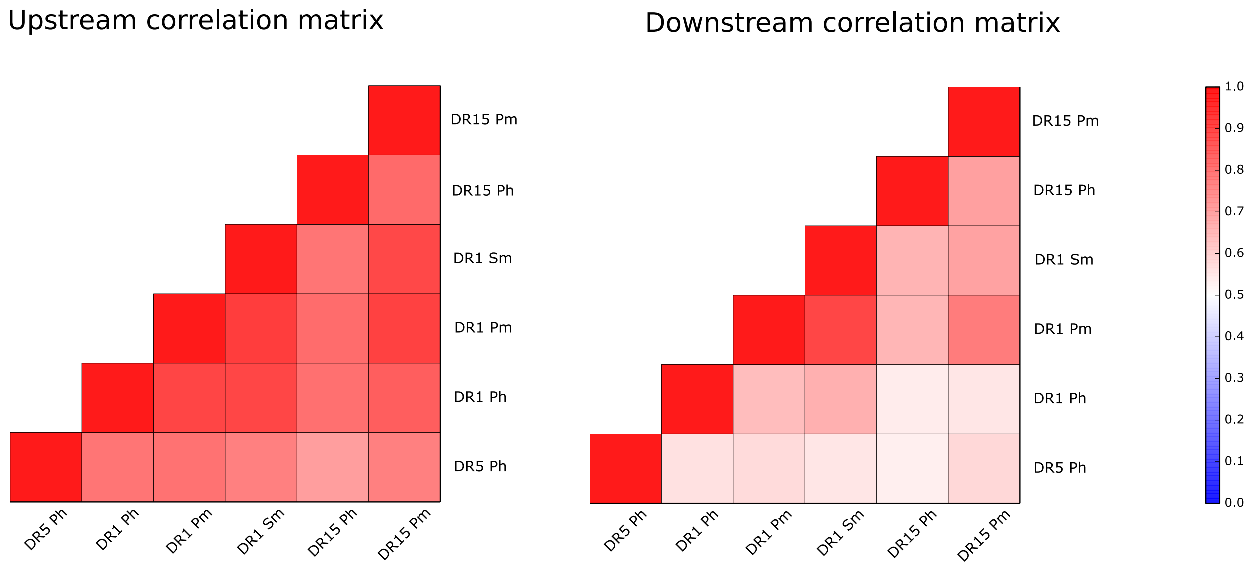


**Figure S6.** Correlation between processing signals found in all six datasets employed in this work, for Upstream and Downstream regions, removing the MS-derived Cysteine enrichment artifact. Each matrix entry displays the Pearson Correlation Coefficient (PCC) value of two datasets under study. A PCC value of one corresponds to a maximum correlation, while a PCC value of zero means no correlation at all. Processing signals used in this figure were generated as explained in Figure 5.

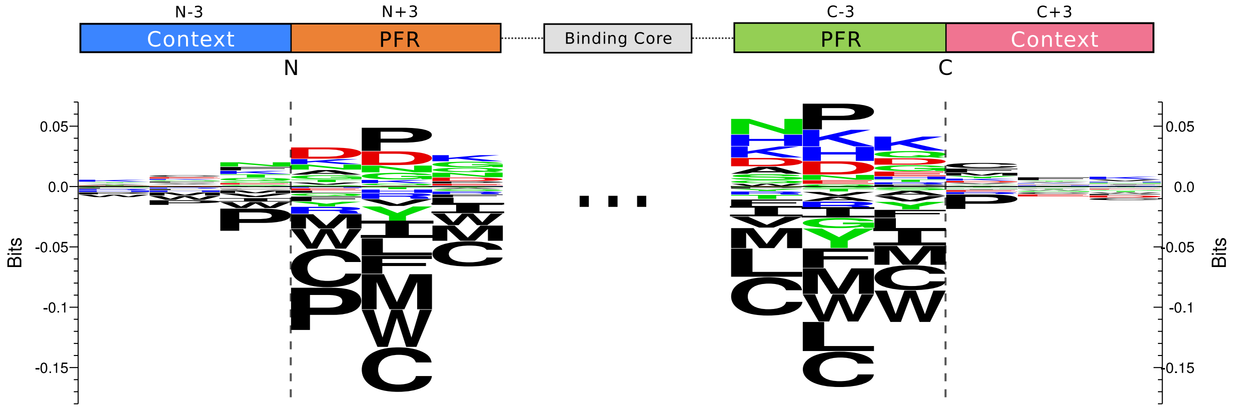


**Figure S7.** Processing signal learned by the NNAlign model trained on DR15 Pm dataset with context information. For each logo, three amino acids of the PFR and context residues neighbouring the N and C terminus are shown. Logos were constructed from the predicted binding cores in the top 1% scoring predictions of 1 million random natural peptides.
